# The Association Between Morningness-Eveningness Preference, Depression, Anxiety and Insomnia Among Chinese Textile Workers With or Without Shift Work

**DOI:** 10.3389/fpsyt.2022.915476

**Published:** 2022-06-30

**Authors:** Jiaqi Jiang, Dongfang Wang, Andrew Scherffius, Dingxuan Chen, Zijuan Ma, Zihao Chen, Yifan Zhang, Qian Yu, Fang Fan

**Affiliations:** ^1^School of Psychology, Centre for Studies of Psychological Applications, Guangdong Key Laboratory of Mental Health and Cognitive Science, Ministry of Education Key Laboratory of Brain Cognition and Educational Science, South China Normal University, Guangzhou, China; ^2^Department of Microbiology and Immunology, Montana State University, Bozeman, MT, United States

**Keywords:** insomnia, anxiety, depression, morningness–eveningness preference, shift work

## Abstract

**Objective:**

Circadian preference and mental health disorders are closely related to insomnia. This study aimed to evaluate insomnia symptoms in textile factory workers with different work schedules, and to investigate the association between insomnia, morningness–eveningness preference, anxiety, and depression.

**Methods:**

A total of 3,883 textile workers were assessed using the 3-items of Pittsburg Sleep Quality Index, Composite Scale of Morningness, Beck Anxiety Inventory, Center for Epidemiologic Studies-Depression Scale, and socio-demographic questionnaires.

**Results:**

The prevalence rate of insomnia in textile workers was 16.7% (*N* = 646), with 49.8% (*N* = 322) were shift workers. Among shift workers (*N* = 1,833), 9.5% had difficulty initiating sleep, and almost 9.0% suffered from early morning awakening, a rate significantly higher than among daytime workers. Logistics regressions revealed that work schedule was insignificantly associated with insomnia. Depression (OR = 1.034, 95% CI = 1.022–1.046) and anxiety (OR = 1.031, 95% CI = 1.018–1.043) positively predicted insomnia, whereas morningness preference (OR = 0.977, 95% CI = 0.960–0.995) decreased the likelihood of insomnia. Furthermore, mediation analysis showed that both anxiety and depression independently mediated the association between circadian preferences and insomnia in textile workers with or without shift work.

**Conclusion:**

This study highlighted the insomnia, depression, and anxiety of textile factory workers in a Chinese textile factory. To improve insomnia symptoms, interventions to promote morningness circadian preference and reduce depressive and anxious symptoms among workers are encouraged.

## Introduction

Insomnia is a widespread and formidable public health concern affecting worldwide from 16.8 to 31% of the general population (1–4). Insomnia is characterized by continuous difficulty in initiating or maintaining sleep and/or early morning awakening (EMA), coupled with related functional impairments like fatigue or difficulty concentrating during the daytime (5). Health risks associated with insomnia include numerous chronic and mental health disorders, including heart disease, metabolic syndrome, obesity, depression, and anxiety (6, 7).

Among shift workers in particular, insomnia has far-reaching repercussions, however sleep-related issues within this demographic have not received considerable attention up to now. Above 25% of the global workforce engages in some form of shift work, and approximately 20–30% of shift workers report insomnia (8, 9). The consequences of such widespread insomnia and other sleep-related problems among shift workers include poor work performance and productivity, as well as accidents and mistakes at work (10–12). Research into the causes of this epidemic suggests that the irregular but inevitable nature of shift work schedule itself is a significant disturbance to circadian rhythms (13, 14). For example, recent findings suggest that shift work leads to short sleep and excessive fatigue due to a disruption of the normal sleep-wake cycle (8, 15). The mechanisms by which insomnia affects workers’ health may be related to disruption of the circadian rhythm.

Circadian rhythm is a specific sleep/wake cycle, regulated by endogenous (e.g., clock genes) and exogenous factors (e.g., light) (16, 17). The various ways in which individuals’ circadian rhythms synchronize to a 24-h cycle are typically divided into three categories, otherwise known as chronotypes: morningness type, eveningness type, and no preference. Individuals with a morningness type wake up and go to sleep earlier and have less variation in sleep time than those who prefer eveningness. Those of the morningness pattern also tend to achieve peak work performance earlier in the morning than those of the eveningness type, who are more efficient later in the day (18). In addition, circadian rhythm interventions targeting maladaptive chronotypes have been used as preventative treatments in a variety of health domains (19, 20). Further, morningness–eveningness preference is closely related to mental health problems (21). Previous study found that eveningness chronotype resulted in decreased melatonin and alterations in diurnal cortisol secretion, which is linked to anxiety disorders generally (22, 23). Importantly, chronotype is due in part to multiple genetic contributions, and there is considerable evidence indicates that eveningness and anxiety may represent a phenotype of a shared underlying genotype (24, 25). Likewise, eveningness is related to cognitive reactivity, which is a vulnerability factor for depression (26). Thus, patients with depressive disorder may have later chronotype. Nevertheless, more research is still required to understand the associations between chronotype, anxiety and depression in Chinese workers.

There are considerable evidences treated eveningness preference, anxiety, and depression as putative risk factors for insomnia. One recent study of 1,323 university students shown that evening-type circadian rhythm significantly predicted insomnia (27). Similarly, an increasing number of studies show that mental health is closely related to insomnia symptoms. For example, evidence has shown that higher levels of depression and anxiety correspond with greater sleep disturbance (28). In a cohort study of elderly Asian subjects, depression and anxiety were found to be associated with sleep-related issues, such as perceived sleep quality, sleep latency, and daytime dysfunction (29). In addition, there is evidence that subjects with anxiety are more likely to develop insomnia than those without anxiety (30). Epidemiological data has also shown that, among DSM-IV mental disorders, organic diseases, and chronic pain, major depressive disorder is the strongest predictor for insomnia (31). However, the mediator roles of depression and anxiety between morningness and eveningness preference and insomnia among workers with or without shift work have only scarcely been investigated.

As a first step toward improving the health and wellbeing of workers, we sought to assess the prevalence of insomnia and the factors influencing insomnia symptoms among workers with or without shift work, and then evaluate the mediator roles of anxiety and depression in the relationship between circadian type and insomnia within our sample. Taken together, we hypothesize that among Chinese textile workers with different work schedules (1) eveningness preference can directly predict insomnia and (2) this relationship is indirectly mediated, in parallel, by anxiety and depression.

## Materials and Methods

### Participants and Procedure

The participants were employees at a textile company in Shandong province, China. Of the 4,177 workers initially surveyed, we excluded 339 participants who (i) finished the questionnaire in less than 15 min (140 participants), (ii) did not complete the entire set of socio-demographic information such as age, gender, phone number, work schedule, height, and weight (128 participants), or (iii) responded with repetitive answers (21 participants). Data from 3,833 participants were included in our final analyses.

This investigation, including the self-report forms distributed to participants online, took place from October 25th to November 15th, 2018. Before initiating this study, permission was obtained from the Ethics Committees of South China Normal University, and each participant signed written informed consent. Participants were reminded of their free right to suspend the study process or to withdraw at any time during the study and that certain compensation would be offered for completion of the questionnaires.

### Measures

#### Socio-Demographic Characteristics

The personal questionnaire collected socio-demographic information about gender, age, height, weight, marital status, residence location, and shift work schedule. Height and weight were used to calculate BMI. As for the shift work system of this factory, the daytime shift is from 8 a.m. to 8 p.m., and the night shift is from 8 p.m. to 8 a.m., with 24 h of rest for employees between each shift. Within the total sample of 3,833 workers, 1,883 participants (49%) had night shift work schedules frequently during the past 6 months, while the remaining 1,950 workers (51%) had normal daytime work schedules. Participants also answered questions about their lifestyle, including weekly frequency of exercise and whether or not they had consumed alcohol and/or smoked within an hour of falling sleep during the past month.

#### Circadian Rhythm

The Composite Scale of Morningness (CSM) was used to assess the patterns of individual circadian rhythm (32). The 13-item scale is designed to measure individual differences in morningness–eveningness preference. The total scores range from 13 (extreme eveningness) to 55 (extreme morningness). And the Chinese version we used has been shown to have adequate psychometric properties (33). The Cronbach’s alpha of CSM was 0.743 in the current sample.

#### Depressive Symptoms

The Center for Epidemiologic Studies-Depression Scale (CES-D) was used to assess the depressive symptoms of participants in the past 2 weeks (34). Twenty items were scored on a 4-point scale ranging from 0 (never) to 3 (always), and a cutoff score of 16 was used to classify a respondent as having symptoms of depression. The CES-D has been widely used in Chinese studies (35). In this study, Cronbach’s α was 0.834.

#### Anxiety Symptoms

The Beck Anxiety Inventory (BAI) was used to measure the severity of anxiety that participants experienced in the past two weeks (36). This 21-item self-report questionnaire ranks questions on a 4-point scale (0 = Not at all, 1 = Mild, 2 = Moderate, 3 = Severe), with a score of 45 or higher regarded as indicative of significant anxiety symptoms. Good retesting reliability and validity for the BAI have been confirmed in Chinese individuals (37). In our study, Cronbach’s alpha for these items was 0.945.

#### Insomnia Symptoms

Three items from the Pittsburg Sleep Quality Index (PSQI) were selected to measure insomnia symptoms (38). These three items were used to examine difficulty in initiating sleep (DIS), difficulty in maintaining sleep (DMS), and EMA among shift workers in the past month (39). Participants indicated the degree to which they agreed with each item on a 5-point scale (1 = never to 5 = almost every day). When three items were rated as “more than three times a week,” this indicated probable and clinically significant insomnia symptoms. Previous studies have demonstrated that the Chinese version of this scale has good reliability and validity (40). Cronbach’s alpha for these three items was 0.784 in the current sample.

### Data Analysis

SPSS 23.0 was used for data evaluation, with the statistical significance threshold determined as *p* < 0.05. No reason for potential multicollinearity of all continuous variables was found, with the variance inflation factor (VIF) of 1.43 and below (41). First, the Welch *t*-test and Chi-square test were employed to examine the associations between insomnia and socio-demographic characteristics (gender, age, BMI, marital status, residence location, smoking, drinking, and exercise), depression, anxiety among shift workers, daytime workers, and the total sample. The Chi-square test was further used to compare different prevalence rates and patterns of insomnia symptoms between worker groups with different work schedules. Next, a binary logistic regression was performed to investigate respective risk or protective factors of insomnia. The strength of each association was estimated by odds ratio (OR) with a 95% confidence interval (CI).

We further explored the relationship between morningness and eveningness preference, depression, anxiety, and insomnia *via* mediation analyses performed using PROCESS (42). A 5,000-time bootstrap bias-corrected and accelerated procedure was implemented for more robust results. Level of statistical significance is determined if certain 95% CI does not contain 0 (43). Demographic information such as age, gender, exercise habits, and residence location were included as covariates in mediation analysis.

## Results

A total of 3,833 workers were invited to participate in the research, 53.4% of whom were males (*N* = 2,048). The age of participants ranged from 17 to 60, and the mean age was 34.45 (SD ± 8.10). Compared with healthy controls, workers with insomnia were significantly more likely to smoke or drink before sleep (22.7 vs. 15.8%; 22.1 vs. 15.8%) and less likely to exercise regularly (14.2 vs. 19.4%). Results also revealed that, among daytime workers, elders, and people who lived in urban areas were more likely to report insomnia; however, these patterns did not hold true among shift workers. Other demographic characteristics were reported in Table 1.

**TABLE 1:** Socio-demographic characteristics and their associations with insomnia symptoms (*n* = 3,883).

Variables	Shift workers *N* = 1,883			Daytime workers *N* = 1,950			Total *N* = 3,833		
			
		Insomnia *N* = 322			Insomnia *N* = 324			Insomnia *N* = 646	
						
	*N* (%)	*N* (%)	*t*/x^2^	*N* (%)	*N* (%)	*t*/x^2^	*N* (%)	*N* (%)	*t*/x^2^
Age			1.036			0.663			1.190
Mean ± SD	33.46 ± 7.40	34.07 ± 7.40		34.43 ± 8.72	34.16 ± 7.72		34.45 ± 8.10	34.11 ± 7.56	
BMI			−1.692			−0.982			−1.890
Mean ± SD	23.29 ± 3.52	23.60 ± 3.44		23.36 ± 3.47	23.53 ± 3.66		23.33 ± 3.49	23.56 ± 3.55	
Gender			0.02			0.16			0.056
Male	1,129 (60.0)	192 (17.0)		919 (47.1)	156 (17.0)		2,048 (53.4)	348 (17.0)	
Female	754 (40.0)	130 (17.2)		1,031 (52.9)	168 (16.3)		1,785 (46.6)	298 (16.2)	
Marital status			1.16			5.8			0.879
Married	1,567 (83.2)	265 (16.9)		1,588 (81.4)	273 (17.2)		3,155 (82.3)	538 (17.1)	
Unmarried/single	264 (14.0)	50 (18.9)		332 (17.0)	43 (13.0)		596 (15.5)	93 (15.6)	
Divorced/widowed	52 (2.8)	7 (13.5)		30 (1.5)	8 (26.7)		82 (2.1)	15 (18.3)	
Residence location			3.544			10.572**			12.03**
Urban	510 (27.1)	92 (18.0)		762 (39.1)	146 (19.2)		1,272 (33.2)	238 (18.7)	
Town	514 (27.3)	98 (19.1)		507 (26.0)	90 (17.8)		1,021 (26.6)	188 (18.4)	
Rural	859 (45.6)	132 (15.4)		680 (34.9)	88 (12.9)		1,539 (40.2)	220 (14.3)	
Smoking			4.138*			16.369***			17.619***
Yes	362 (19.2)	75 (20.7)		251 (12.9)	64 (25.5)		613 (16.0)	139 (22.7)	
No	1,521 (80.8)	247 (16.2)		1,689 (87.1)	260 (15.4)		3,219 (84.0)	507 (15.8)	
Drinking			7.733**			7.592***			14.529***
Yes	342 (18.2)	76 (22.2)		283 (14.5)	63 (22.2)		625 (16.3)	138 (22.1)	
No	1,541 (81.8)	246 (16.0)		1,666 (85.5)	261 (15.7)		3,207 (83.7)	508 (15.8)	
Exercise weekly			0.730**			8.006**			18.687***
Yes	899 (47.7)	127 (14.1)		970 (49.7)	138 (14.2)		1,869 (48.8)	265 (14.2)	
No	984 (52.3)	195 (19.8)		979 (50.2)	186 (19.0)		1,963 (51.2)	381 (19.4)	
Depression^a^			59.760^***^			63.569***			123.173***
Yes	489 (26.0)	139 (28.4)		421 (21.6)	124 (29.5)		910 (23.8)	263 (28.9)	
No	1,394 (74.0)	183 (13.1)		1,527 (78.4)	200 (13.1)		2,920 (76.2)	383 (13.1)	
Anxiety^b^			26.170***			35.358***			55.636***
Yes	153 (8.1)	49 (32.0)		122 (6.3)	44 (36.1)		271 (7.1)	90 (33.2)	
No	1,730 (91.9)	273 (15.8)		1,825 (93.6)	280 (15.3)		3,562 (92.9)	556 (15.6)	

*p < 0.05, **p < 0.01, ***p < 0.001.

*^a^The cut-off score is 16. ^b^The cut-off score is 45.*

As shown in Table 2, approximately 16.7% (*N* = 646) of workers within the total sample reported insomnia, with 49.8% (*N* = 322) being shift workers. The proportion of insomnia showed no significant difference between shift workers and daytime workers. The proportion of workers who had DIS and experienced EMA was higher among shift workers than among daytime workers; however, the proportion of those who had DMS was not significantly different between these groups.

**TABLE 2:** The prevalence rate and patterns of insomnia symptoms between shift workers (*n* = 1,833) and daytime workers (*n* = 1,950).

Variables	Shift workers *N* = 1,883		Daytime workers *N* = 1,950		χ^2^	Total *N* = 3,833	
				
	*N*	(%)	*N*	(%)		*N*	(%)
Insomnia	322	17.1	324	16.6	0.155	646	16.7
DIS					4.763*		
Yes	178	9.5	146	7.5		324	8.5
No	1,705	90.5	1,803	92.5		1,705	91.5
DMS					0.626		
Yes	191	10.1	213	10.9		404	10.5
No	1,692	89.9	1,736	89.1		3,428	89.5
EMA					9.609**		
Yes	170	9	124	6.4		294	7.7
No	1,713	91	1,825	93.6		3,538	92.3

*p < 0.05, **p < 0.01.

*DIS, difficulty in initiating sleep; DMS, difficulty in maintaining sleep; EMA, early morning awakening.*

Results of the binary logistic regression analysis on factors associated with insomnia in the whole sample are summarized in Table 3. These results indicate that depression (OR = 1.034, 95% CI = 1.022–1.046) and anxiety (OR = 1.031, 95% CI = 1.018–1.043) were potential risk factors for insomnia, while living in a rural area (OR = 0.678, 95% CI = 0.548–0.839), weekly exercise (OR = 0.706, 95% CI = 0.590–0.845), and morningness preference (OR = 0.977, 95% CI = 0.960–0.995) served as protective factors against insomnia. The adjusted *R* square for this model was 8.4%.

**TABLE 3:** Binary regression analyses of the factors associated with insomnia.

Variable	Wald	OR	95% CI	*p*
Residence location^#^ (town)	0.300	0.940	0.753–1.173	0.584
Residence location^#^ (rural)	12.743	0.678	0.548–0.839	<0.001
Smoking (yes)	2.574	1.226	0.956–1.573	0.109
Drinking (yes)	0.631	1.105	0.864–1.413	0.427
Exercise weekly (yes)	14.476	0.706	0.590–0.845	<0.001
Work schedule (shift work)	0.001	0.997	0.835–1.190	0.972
Age	1.390	1.007	0.995–1.019	0.238
CES-D score	31.385	1.034	1.022–1.046	<0.001
BAI score	23.205	1.031	1.018–1.043	<0.001
CSM score	6.505	0.977	0.960–0.995	0.011

*CES-D, Center for Epidemiologic Studies-Depression Scale; BAI, Beck Anxiety Inventory; CSM, Composite Scale of Morningness; OR, odds ratio; CI, confidence interval.*

*^#^Urban as Ref.*

**FIGURE 1:**
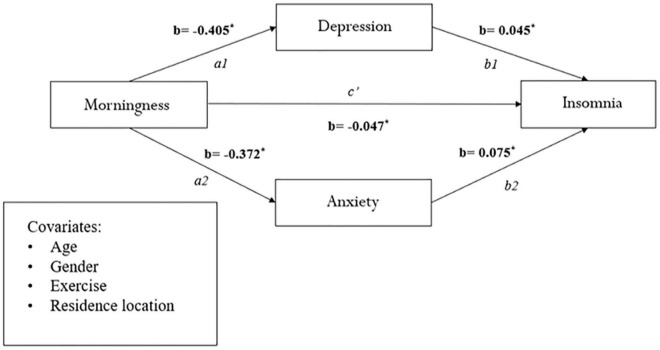
Mediation analysis between morningness and eveningness preference and insomnia in the whole sample (*n* = 3,833). *p < 0.001.

**FIGURE 2:**
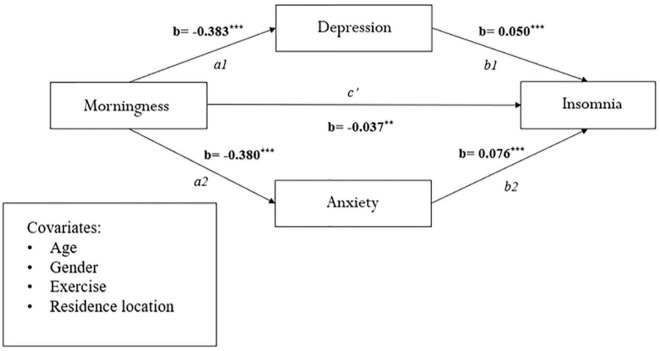
Mediation analysis between morningness and eveningness preference and insomnia in shift workers (*n* = 1,833). **p < 0.01, ***p < 0.001.

**FIGURE 3:**
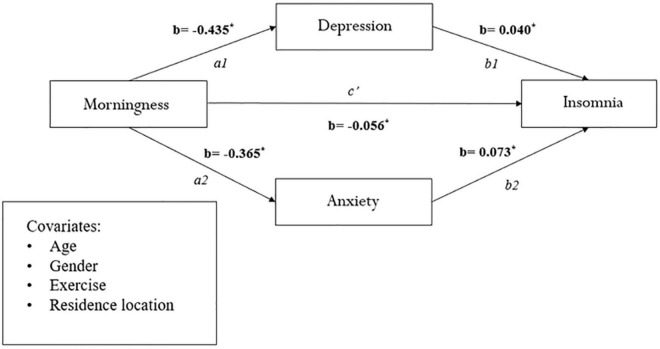
Mediation analysis between morningness and eveningness preference and insomnia in daytime workers (*n* = 1,950). *p < 0.001.

Figure 1, which uses the bootstrapping method, portrays the mediating role of depression and anxiety on the relationship between morningness preference and insomnia, in parallel, across the whole sample [c1: β = −0.018; CI (−0.025, −0.012); c2: β = −0.028; CI (−0.035, −0.021)]. These results explain the 27.7 and 37.3% variances, respectively. We also tested the significance of morningness preference on insomnia as mediated by depression and anxiety in the two worker groups—shift workers and daytime workers, and similar results were obtained (see Figures 2, 3).

## Discussion

To our knowledge, this is the first study to investigate the relationship between circadian preferences and insomnia in Chinese textile workers with different work schedules. Our findings suggested that morningness, anxiety, and depression were risk factors for insomnia. In particular, morningness preference was associated with lower risk of insomnia and was independently mediated by anxiety/depression among shift workers, daytime workers, and the whole worker sample, respectively.

### Insomnia in Textile Workers With or Without Shift Work

Among 3,833 workers we recruited, the prevalence rates of insomnia were 16.7% (*N* = 646), and the incidence of insomnia among shift workers and daytime workers was 17.1% (*N* = 178) and 16.6% (*N* = 324), respectively. Congruent with previous research, these findings emphasize that sleep problems are a common concern for workers (11).

Other than traditional daytime and weekdays work schedule, some industries need shift workers to operate and provide services during non-mainstream working hours such as holidays or evenings. In the shift workers sample, around 9.5% had DIS, and almost 9.0% suffered from EMA. These rates were significantly higher than those found in daytime workers. These results are consistent with previous research that suggests (1) sleep disturbances among shift workers often include difficulties falling asleep and (2) daytime sleep latency occurring after a night shift decreases by an average of 1 to 2 h (16). This common phenomenon in shift workers can be attributed to the conflict between the irregular sleep/wake times and the individual’s circadian system due to the non-conventional work schedule (44).

### Influential Factors of Insomnia Among Workers

Our study revealed several risk factors for, and factors protecting against, insomnia among textile factory workers. In particular, morningness preference was found to be a strong protective factor against insomnia. Consistent with previous research, people with eveningness-preference have poorer sleep hygiene practices, meaning they tend to engage in environmental and behavioral habits that lower sleep quality (e.g., drinking coffee or alcohol before sleep, irregular sleep time, improper temperature or light in the bedroom, etc.), and thus have more insomnia symptoms (45). Prior studies have also found that patients with eveningness preference showed poorer response to classic insomnia treatments, thereby suggesting that chronotype might be related to the development of psychiatric diseases through distinct mechanisms (46–48).

In accordance with prior studies, the present findings indicate that higher levels of depression and anxiety symptoms are risk factors for developing insomnia (49, 50). Carskadon has also suggested that psychological vulnerabilities may interact with physiological maturation, thereby causing insomnia (51). Our results may be explained, in part, by the cognitive model of insomnia proposed by Harvey et al. (52). According to this theory, worry and ruminating thoughts during the daytime trigger selective attention to sleep-related threat cues, and may further exacerbate anxiety and depression, contributing to insomnia.

A final major takeaway from our study is that insomnia was not significantly affected by different shift work systems. However, some study has shown an opposite result that shift workers always report more sleep disturbances than day workers, and are explained mainly by the fact that the work schedule is out of phase with the endogenous circadian rhythms (53, 54). In accordance with prior evidences, we speculated that such inconsistency may be due to variations in the nature of the work and corresponding individual vulnerability in selected shift-work samples (55). The rotating schedules in the textile factory under study often allow 24 h of free time before starting the new shift, thereby providing more opportunities for rest and sleep. In future studies, it would be valuable to compare these findings with those collected by workers at other factories with different schedules. Comparing these data, we may find that the impact of shift work on insomnia is eased to a certain extent by regular and adequate breaks during and between shifts.

### The Mediating Effects of Anxiety/Depression

Consistent with previous studies, our mediation analysis showed that morningness preference may decrease the likelihood of insomnia by decreasing anxiety among workers (56, 57). Genetic evidence indicates that the circadian-related genes may play an important role in eveningness and anxious traits (58). In addition, this result might be explained by the “internalization of conflicts” model of insomnia proposed by Kales et al. (59). According to this theory, conflict-internalization resulting from affective states leads to physiological hyperarousal and contributes to the co-occurrence of insomnia.

In addition to anxiety, we found that depression was an effective mediator of the impact of morningness preferences on insomnia. Previous research has indicated that dopamine, a neurotransmitter associated with depression, may be linked to insomnia and eveningness preference (60, 61). Another possible explanation for this result is that people with eveningness preference tend to have more sleep-related dysfunctional cognitions (e.g., rumination and overly worried), and thus lead to more severe insomnia symptoms (62).

In summary, both anxiety and depression independently mediated the association between circadian preferences and insomnia, suggesting that individuals with morningness preference tend to have less anxiety and depression, thereby decreasing their insomnia. Our findings should encourage clinicians to assess the level of depression and anxiety in frontline workers with eveningness preference to identify their increased risk of insomnia.

### Limitations

There are several limitations to this study. First, this research only recruited workers in a single textile factory. Stronger statistical data and more confident conclusions could be attained if different occupational groups are recruited to future studies replicating our design. Another limitation of our study was its cross-sectional method, which requires caution when interpreting the cause-effect connection between variables. Previous studies indicated that the relationship between chronotype and insomnia, as well as the relationship between insomnia and anxiety/depression could be bidirectional (63, 64). While the current study has only tentatively explored one of possible directions of their associations. Therefore, longitudinal studies in the future would be needed to better understand the relationship among morningness–eveningness preference, insomnia, anxiety, and depression. Finally, we acknowledge these analyses may be limited due to the preliminary concurrent validity analyses, which were based on self-report questionnaires rather than clinical interviews, and the presence of other sleep disorders or other neurological comorbid conditions should be considered.

## Conclusion

This study found that morningness preference was sequentially associated with decreased depression symptoms and anxiety symptoms, whether with or without shift work, which led to lower self-reported insomnia. These findings both confirm and extend previous studies, highlighting the mediator roles of depression and anxiety in how circadian rhythm influences vulnerability to insomnia among textile workers. They also suggest the need for continued study of sleep-wake rhythm interventions, such as bright light therapy and melatonin supplements, to improve workers’ sleep schedules. Finally, relevant mental health promotion services, such as mindfulness-based interventions and cognitive control strategies, should be utilized to improve workers’ sleep habits and mental health.

## Data Availability Statement

The original contributions presented in this study are included in the article/supplementary material, further inquiries can be directed to the corresponding author.

## Ethics Statement

Ethical approval was obtained from the Human Research Ethics Committee of South China Normal University, Guangzhou, China.

## Author Contributions

JJ and DW: writing—original draft preparation, writing—review and editing, methodology, and formal analysis. AS: visualization, and writing—review and editing. JJ, DW, DC, ZM, ZC, YZ, and QY: investigation and data curation. FF: supervision, resources, project administration, and funding acquisition. All authors contributed to the article and approved the submitted version.

## Conflict of Interest

The authors declare that the research was conducted in the absence of any commercial or financial relationships that could be construed as a potential conflict of interest.

## Publisher’s Note

All claims expressed in this article are solely those of the authors and do not necessarily represent those of their affiliated organizations, or those of the publisher, the editors and the reviewers. Any product that may be evaluated in this article, or claim that may be made by its manufacturer, is not guaranteed or endorsed by the publisher.
